# Long-term ethanol exposure: Temporal pattern of microRNA expression and associated mRNA gene networks in mouse brain

**DOI:** 10.1371/journal.pone.0190841

**Published:** 2018-01-09

**Authors:** Elizabeth A. Osterndorff-Kahanek, Gayatri R. Tiwari, Marcelo F. Lopez, Howard C. Becker, R. Adron Harris, R. Dayne Mayfield

**Affiliations:** 1 Waggoner Center for Alcohol and Addiction Research, University of Texas at Austin, Austin, Texas, United States of America; 2 Department of Psychiatry and Behavioral Sciences, Medical University of South Carolina, Charleston, South Carolina, United States of America; Oregon Health and Science University, UNITED STATES

## Abstract

Long-term alcohol use can result in lasting changes in brain function, ultimately leading to alcohol dependence. These functional alterations arise from dysregulation of complex gene networks, and growing evidence implicates microRNAs as key regulators of these networks. We examined time- and brain region-dependent changes in microRNA expression after chronic intermittent ethanol (CIE) exposure in C57BL/6J mice. Animals were sacrificed at 0, 8, and 120h following the last exposure to four weekly cycles of CIE vapor and we measured microRNA expression in prefrontal cortex (PFC), nucleus accumbens (NAC), and amygdala (AMY). The number of detected (395–419) and differentially expressed (DE, 42–47) microRNAs was similar within each brain region. However, the DE microRNAs were distinct among brain regions and across time within each brain region. DE microRNAs were linked with their DE mRNA targets across each brain region. In all brain regions, the greatest number of DE mRNA targets occurred at the 0 or 8h time points and these changes were associated with microRNAs DE at 0 or 8h. Two separate approaches (discrete temporal association and hierarchical clustering) were combined with pathway analysis to further characterize the temporal relationships between DE microRNAs and their 120h DE targets. We focused on targets dysregulated at 120h as this time point represents a state of protracted withdrawal known to promote an increase in subsequent ethanol consumption. Discrete temporal association analysis identified networks with highly connected genes including ERK1/2 (mouse equivalent Mapk3, Mapk1), Bcl2 (in AMY networks) and Srf (in PFC networks). Similarly, the cluster-based analysis identified hub genes that include Bcl2 (in AMY networks) and Srf in PFC networks, demonstrating robust microRNA-mRNA network alterations in response to CIE exposure. In contrast, datasets utilizing targets from 0 and 8h microRNAs identified NF-kB-centered networks (in NAC and PFC), and Smad3-centered networks (in AMY). These results demonstrate that CIE exposure results in dynamic and complex temporal changes in microRNA-mRNA gene network structure.

## Introduction

Excessive, chronic alcohol use can evoke persistent alterations in brain function that result in alcohol dependence [1, 2]. Such alterations involve complex gene networks that span multiple brain regions [3], and there is growing evidence that microRNAs may play an important regulatory role in alcohol’s effects on brain function [4–7]. MicroRNAs are short, non-coding RNAs that utilize sequence complementarity to bind RNA transcripts, thus modifying their expression [8]. A single microRNA can simultaneously alter expression of numerous genes, while multiple microRNAs can act coordinately to precisely control the expression of a single gene [7, 9].

Studies have implicated microRNAs in both human [10–13] and animal models of addiction [7, 9, 14–19]. For example, Lewohl et al. [10] have shown that numerous microRNAs are upregulated in post-mortem frontal cortex of human alcoholics and similarly, microRNA expression is modified in cortices of ethanol-dependent mice [15]. MicroRNAs may be responsible for the ethanol-induced response of addiction-associated signaling molecules such as the D1 dopamine receptor, *Drd1* [20] and brain-derived neurotrophic factor, *Bdnf* [17, 21]. Importantly, several studies have demonstrated perturbations of miRNA-mRNA networks as a result of ethanol exposure [7, 9, 11] but to our knowledge, none has directly evaluated the temporal nature of such networks.

The chronic intermittent ethanol vapor (CIE) paradigm is known to increase voluntary ethanol consumption in rodents and is considered to be a model of ethanol dependence [22–24]. Recently, Smith and colleagues [25] conducted a time-course experiment examining gene expression in five brain regions following CIE vapor and identified a PFC gene co-expression module enriched with predicted binding sites for several microRNAs that may regulate genes within the module, but microRNA levels were not directly assessed. Previously, we used the CIE model to investigate gene expression changes in three different brain regions (amygdala, AMY; nucleus accumbens, NAC; prefrontal cortex, PFC) at 0, 8 and 120h following exposure to 4 cycles of CIE vapor. These time points represent distinct responses to ethanol exposure, including intoxication (0h), withdrawal (8h) and protracted abstinence (120h). In all brain regions, we identified overlapping time-based gene clusters and gene co-expression modules that shared cell-type specific signatures, perhaps suggesting a common regulatory mechanism. We hypothesized that CIE-induced alterations in microRNA expression could be linked with some of the gene expression changes, enabling us to define the role of microRNAs in gene network changes produced by CIE exposure and withdrawal.

We report microRNA expression changes in three brain regions (AMY, NAC and PFC) at three time points following 4 cycles of CIE vapor in mice. For each brain region, differentially expressed (DE) microRNAs were paired with their putative mRNA targets DE in our previous study [26]. Importantly, the mRNA and microRNA expression profiles in the current study were obtained from the same samples used in our earlier study (24). Two separate approaches (discrete temporal association, hierarchical clustering) were combined with pathway analysis to further characterize the temporal relationships between DE microRNAs and their 120h DE targets. Robust microRNA-mRNA network alterations in response to CIE exposure were identified demonstrating that CIE exposure results in complex temporal changes in microRNA-mRNA gene network structure.

## Materials and methods

### Ethics statement

All procedures were approved by the Medical University of South Carolina Institutional Animal Care and Use Committee and adhered to NIH Guidelines. The Medical University of South Carolina animal facility is accredited by the Association for Assessment and Accreditation of Laboratory Animal Care.

### Animals and chronic ethanol inhalation procedure

Adult male C57BL/6 mice, purchased from Jackson Laboratories (Bar Harbor, ME), were used in this study. Mice were individually housed under a 12-hr light/dark cycle (lights on at 4:00 AM) in a temperature- and humidity controlled- animal facility. The animals had free access to food (Teklad rodent diet) and water throughout the experiment. The study began after an acclimation period (one week) and mice were monitored daily by the animal facilities staff and the research technicians.

Chronic ethanol inhalation procedures, tissue harvest, and brain dissection methods were as previously described [26–29]. Briefly, chronic intermittent ethanol vapor exposure (or air) was delivered in Plexiglas inhalation chambers to drug-naïve C57BL/6J (B6) male mice (8 treated and 8 controls per group). Ethanol treatments were performed in the laboratory of Dr. H.C. Becker (Medical University of South Carolina, Charleston, SC, USA). Mice were administered alcohol (1.6 g/kg; i.p.) and the alcohol dehydrogenase inhibitor pyrazole (1 mmol/kg; i.p.) prior to vapor ethanol exposure in inhalation chambers. Control subjects received pyrazole injections in saline and received similar daily handling. Chamber ethanol concentrations were monitored daily to induce stable blood ethanol concentrations within the range of 180–200 mg/dl. Ethanol was administered 16 h/day in 4 weekly cycles and alternated with 1 week in between cycles in which the mice were left undisturbed (mimicking drinking weeks). Animals were sacrificed at 3 time points: 0-, 8- and 120-hours following the last ethanol vapor or air treatment. RNA purification, quantification and quality assessment were performed as in [26]. Briefly, total RNAs were purified using the MagMax 96 for Microarrays (Ambion, Austin, TX) kit using a modified protocol that allowed recovery of small and microRNAs, as well as total RNA. RNAs were quantified on a NanoDrop 1000 spectrophotometer (Thermo Fisher Scientific, Inc., Rockland, IL) and RNA quality was assessed on either the Agilent 2100 Bioanalyzer or 2200 TapeStation (Agilent Technologies, Santa Clara, CA). Recovery of low molecular weight RNAs (i.e., <200 nucleotides) was confirmed by presence of a diffuse band/peak around 100 nucleotides. (These are the same RNAs prepared in [26]; the protocol modification was inadvertently omitted from that publication.)

### MicroRNA array analysis

Total RNAs from amygdala (AMY), nucleus accumbens (combined core and shell, NAC) and prefrontal cortex (PFC) were shipped to the Molecular Genomics Core Facility at Moffitt Cancer Center (Tampa, FL). Samples were biotin-labeled using the FlashTag Biotin HSR RNA Labeling Kit (Affymetrix, Santa Clara, CA) and hybridized to GeneChip miRNA 3.0 arrays (Affymetrix, Santa Clara, CA) according to manufacturer instructions. This platform used annotations from miRbase version 17 and contained probe sets for over 19,000 mature microRNAs from 153 species. Transcript abundance was measured by fluorescent intensity after scanning with the Affymetrix GCS3000 scanner and generation of cel files with Affymetrix AGCC v3 software. MicroRNA array data have been submitted to the NCBI Gene Expression Omnibus (GEO) (http://www.ncbi.nlm.nih.gov/geo/) under accession number GSE90608. Arrays were hybridized with material from a single animal (no pooling); thus, 144 samples (16 animals x 3 tissues x 3 time points) were profiled.

### Statistics and bioinformatics

Data were analyzed using open source software packages from Bioconductor (http://bioconductor.org) designed for the statistical language R (http://www.r-project.org) and Microsoft Excel unless otherwise noted. Data from each brain region were analyzed independently. Gene Expression Console (version 1.4, Affymetrix, Santa Clara, CA) was employed for data preprocessing (detection above background, DABG [30], and robust multichip analysis, RMA [31]) and identification of sample outliers using all mouse probesets. Five samples (one AMY 120h control, one PFC 0h control, two NAC 8h controls and one NAC 0h CIE-treated) were identified as outliers using Gene Expression Console and were removed. Data were filtered to include only mature mouse microRNAs with a detection p value < 0.06 on 80% of arrays. Mouse microRNAs were updated to miRbase version 21 annotations using miRbaseTracker [32]. Differential expression analysis for each time point was conducted using empirical Bayes moderated t-statistics from the Bioconductor package limma [33] to compare treated and control mice. MicroRNAs were considered DE at a nominal value ≤ 0.05. A nominal, rather than FDR-corrected, p value was utilized to preserve as much DE information as possible for our subsequent systems-level analyses as use of an overly-stringent statistical cutoff would cause us to lose valuable network relationships. Although depleted RNA samples precluded confirmation of our results by RT-qPCR, we have used RT-qPCR to validate this microRNA array platform in a previous publication [7]. In that study, RT-qPCR confirmed microRNA expression changes for six different microRNA families in both mouse and human brain tissue.

Two separate approaches (discrete temporal association, hierarchical clustering) were used to uncover the temporal relationship(s) between microRNAs and their downstream targets in each brain region. Both methods used the "microRNA target filter" utility in Ingenuity Pathway Analysis (IPA, September 2015 release, Qiagen Redwood City, www.qiagen.com/ingenuity) to associate microRNAs with their previously reported DE mRNA targets [26]. It should be noted that microRNAs and mRNAs were isolated from the same samples. Data from mRNA analyses were deposited in the NCBI Gene Expression Omnibus (GEO) under accession number GSE 60676. Only experimentally observed microRNA-target interactions and those predicted with high confidence were used. IPA assigns "high confidence" to interactions involving a conserved or highly conserved microRNA as defined by TargetScan [34–37] and at least one conserved site on the targeted sequence or having a TargetScan total context score of -0.4 or less.

**Fig 1:**
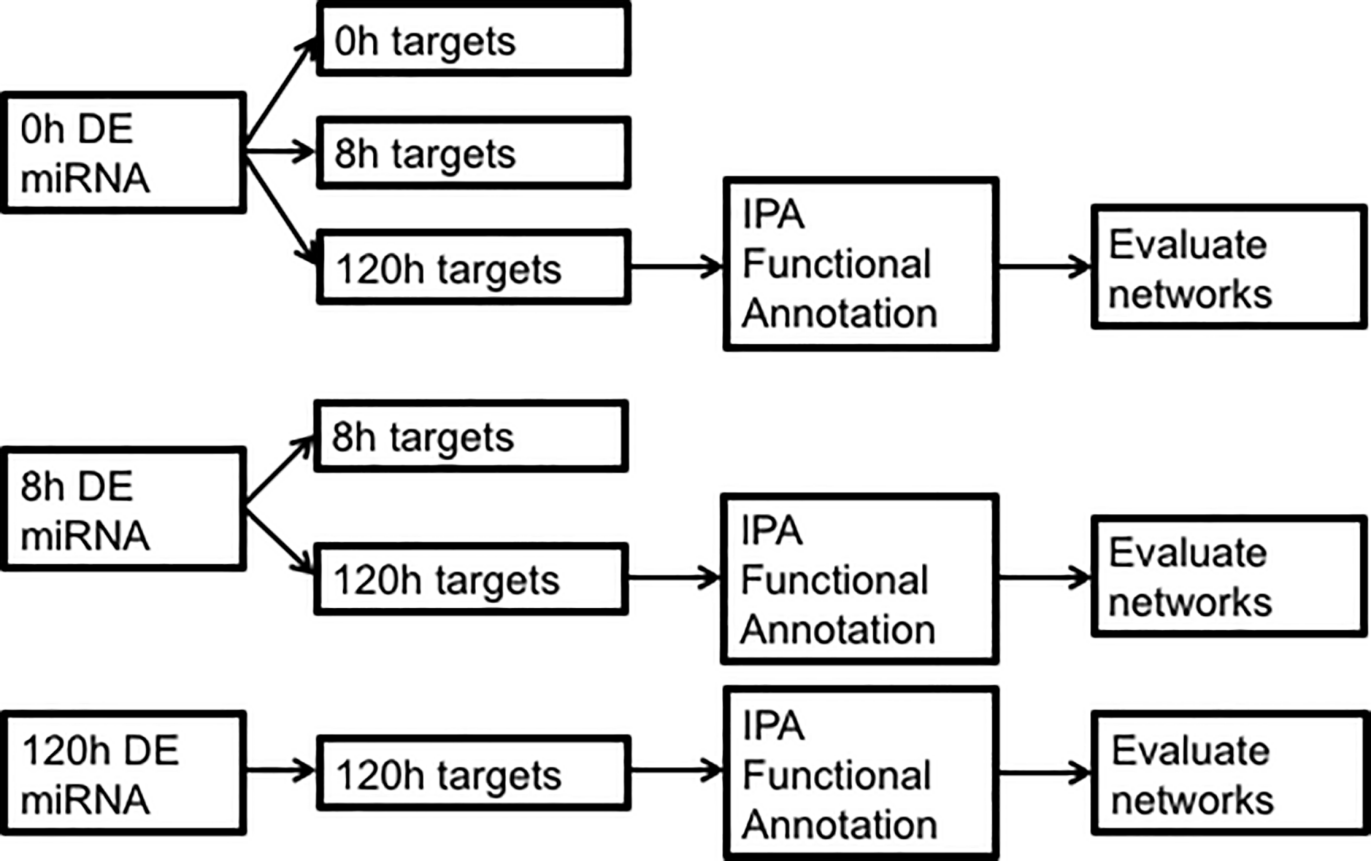
Overview of procedure used to pair DE microRNAs with DE targets and analyses performed on resulting datasets.

In the first discrete temporal association analysis, differentially expressed microRNAs from each time point were paired with differentially expressed targets (predicted and validated) at each time point. To maintain consistency with previous analyses, we used linear fold changes and an FDR cutoff of 0.05 for targets DE at 0 or 8h and a nominal p value cutoff of 0.05 for targets DE at 120h. (Previously, these thresholds were chosen in order to maximize the amount of information we could extract from the data. Since the 120h time point did not produce any DE mRNAs after correction for multiple comparisons, implementing an FDR cutoff for all time points would have excluded this entire time point from analysis. Thus, we chose to utilize an FDR threshold where possible and a nominal p value otherwise. Although this is a pitfall in our study, we reasoned that it would allow us to utilize all of our data while maintaining as much rigor in the analysis as possible.) Only microRNA-mRNA associations consistent with changes in microRNA proceeding changes in mRNA were considered. For example, microRNAs DE at 8h were paired with targets from the 8 and 120h time points but not the 0h time point. In all, six paired datasets were created for each brain region (S1 Table). Paired data sets are codified as TimePoint_DEmiR/TimePoint_DEtargets. For example, "0hDEmiR/120hDEtargets" specifies the microRNAs DE at 0h and their targets DE at 120h. We were particularly interested in genes dysregulated at 120h, which represents a state of extended withdrawal, as a result of microRNAs dysregulated at 0h (intoxication) and 8h (withdrawal). Thus, the datasets representing these temporal associations were evaluated with the "Core Analysis" option in IPA using minimum p value to resolve duplicate probes and Illumina MouseRef-8 version 2.0 as the reference set. Fig 1 provides a graphic overview of this method.

An alternative approach, hierarchical clustering, used centered and scaled log ratios of DE microRNAs from all time points per brain region with the package "clValid" (version 0.606) [38]. This R package was used to compare multiple algorithms simultaneously to identify the best clustering approach and the optimal number of clusters. Clustered microRNAs were plotted with R package "MmPalateMiRNA" [39] with scaled log-ratios vs time. Clustering was visualized using GraphPad Prism version 6.07 for Windows, GraphPad Software, La Jolla California USA, www.graphpad.com. The number of unique, non-overlapping expression patterns indicates the number of assigned clusters for each brain region. For each identified microRNA cluster, IPA was used to construct a network relating the microRNAs with their DE gene targets reported in [26] using the same statistical significance thresholds as described above. First, microRNAs in each cluster and their 120h DE targets were added to a new pathway in IPA, and then related and connected using the Ingenuity Knowledge Base. The "grow" tool was used sequentially to add the ten 0h DE targets, and then the ten 8h DE targets, with the highest connectivity to the network, connecting all molecules after each addition. Default settings were used for all IPA pathway functions.

## Results

### MicroRNA descriptive statistics

**Fig 2:**
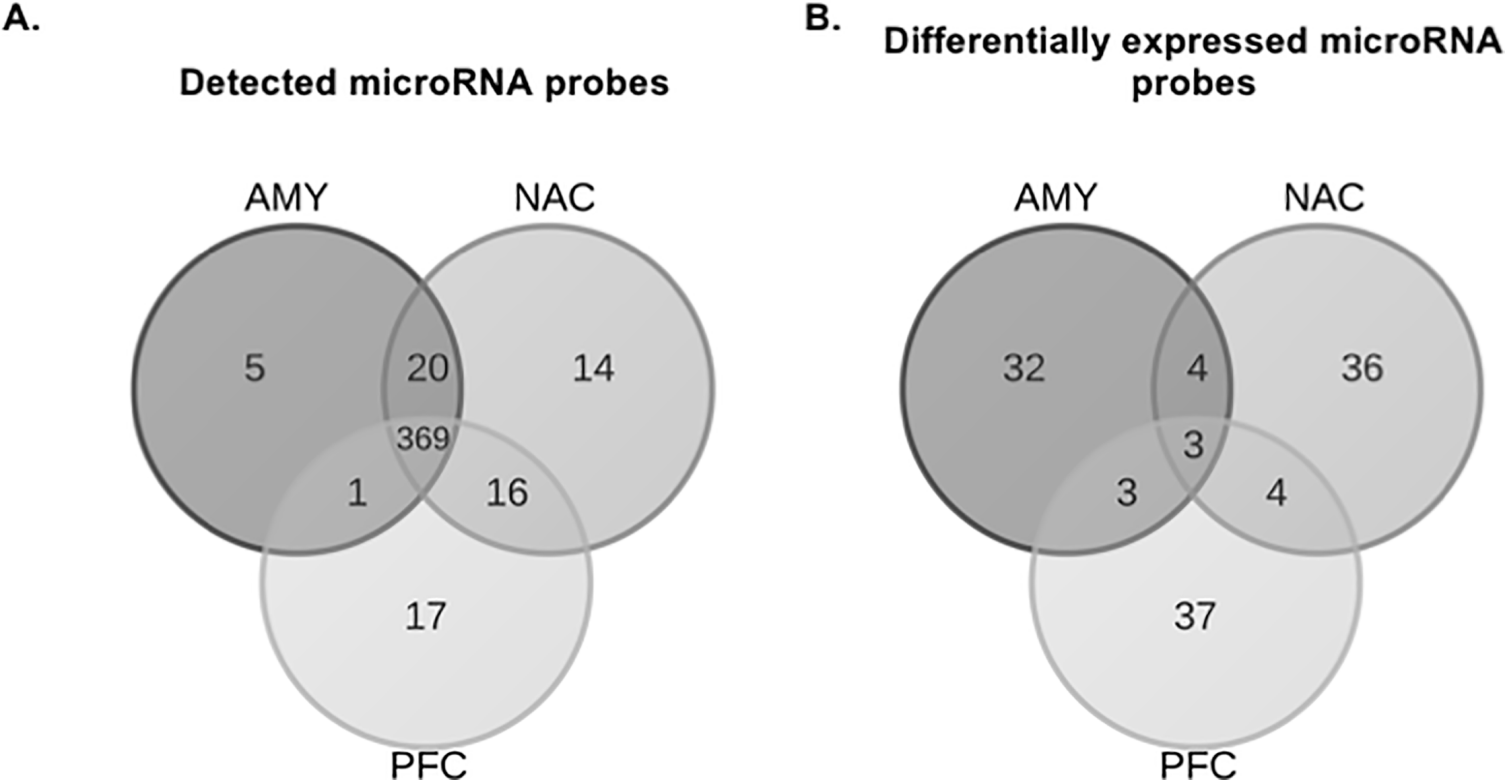
Overlap of probesets among brain regions. Data from all time points were combined for each brain region. Panel A shows overlap of all probes detected within each brain region. Panel B shows overlap of all probes DE (p ≤ 0.05) within each brain region.

**Fig 3:**
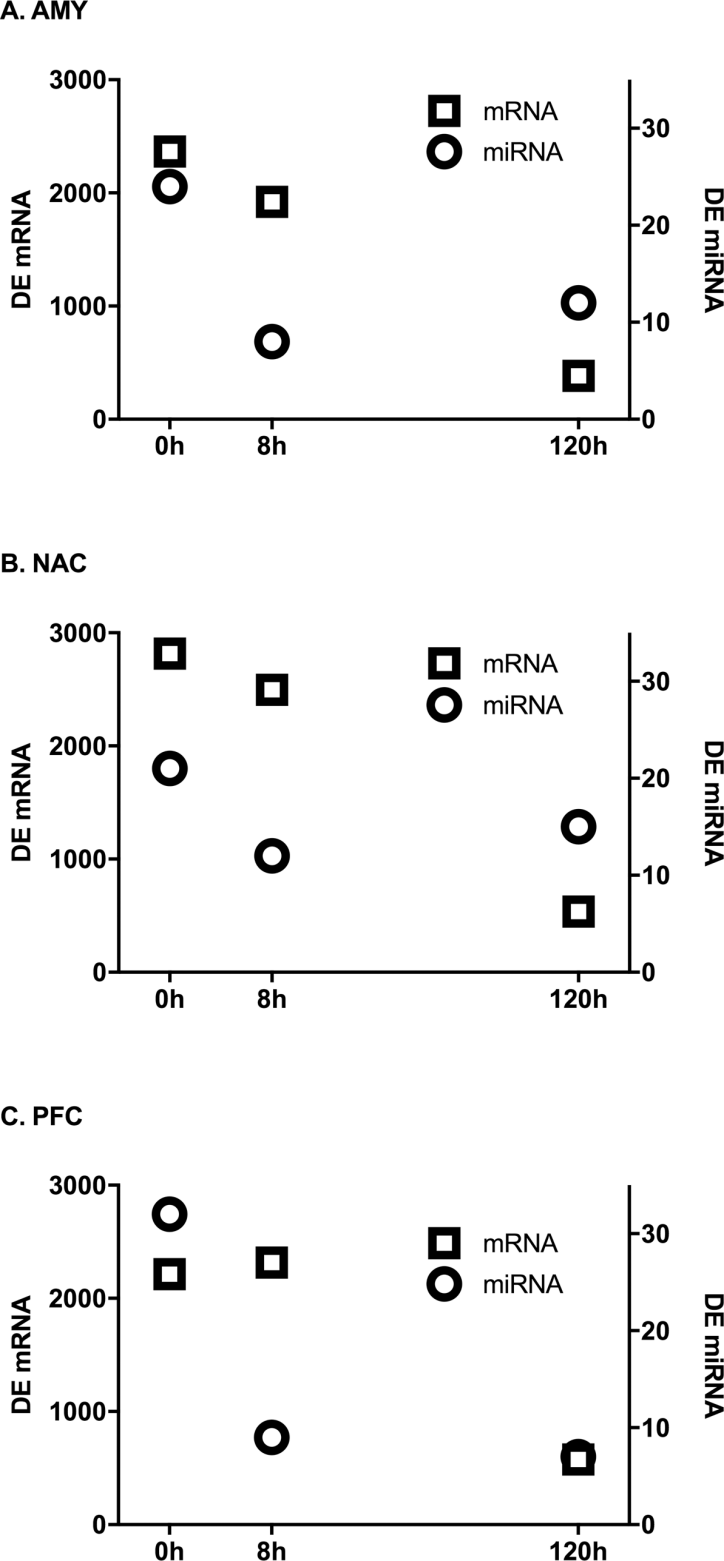
Numbers of microRNAs and mRNAs (from[26]) DE at each time point. Circles represent microRNAs, squares represent genes. Genes at 0 and 8h were considered DE at an FDR of 0.05. MicroRNAs and 120h mRNAs were considered DE at a nominal value of 0.05.

MicroRNAs were profiled in AMY, NAC and PFC at three different time points (0, 8 and 120h) after ethanol vapor treatment. After preprocessing, both the number of detected (395–419) and DE (42–47) microRNA probesets was similar within each brain region. Despite most of the detected microRNAs being expressed in all brain regions, the DE probes were distinct for each brain region (Fig 2). There was little overlap of DE microRNAs across time within each brain region (S1 Fig). MicroRNAs that were uniquely detected and DE in each brain region are shown in Table 1. Only three microRNAs were DE in all three brain regions (Table 2). MicroRNAs and mRNAs exhibited different temporal patterns of dysregulation (Fig 3). AMY and NAC showed very similar temporal profiles with the number of DE mRNAs decreasing over time, while DE microRNAs decreased from 0 to 8 hours and then rebounded at 120h. The temporal pattern of expression was unique in PFC because the number of DE microRNAs decreased as a function of time, and the number of DE mRNAs peaked at 8h, then declined at 120h.

**Table 1:** MicroRNAs uniquely detected in each brain region (included in Fig 2A).

Unique to AMY	Unique to NAC	Unique to PFC
miR-136-3p	miR-122-5p	miR-135a-1-3p
miR-190a-3p	miR-1298-5p	miR-141-3p^@^
miR-21a-5p	miR-193a-3p	miR-200a-3p^@^
miR-331-5p	miR-206-3p	miR-141-5p
miR-34b-3p	miR-297a-5p	miR-182-5p
	miR-3068-5p	miR-183-3p
	miR-339-3p	miR-183-5p
	miR-344d-3-5p	miR-1934-3p
	miR-379-3p	miR-199b-3p
	miR-5113	miR-200a-5p^$^
	miR-669a-3p^&^	miR-200b-5p^$^
	miR-669o-3p^&^	miR-200b-3p^#^
	miR-673-3p	miR-200c-3p^#^
	miR-764-5p	miR-429-3p^#^
		miR-214-3p
		miR-27b-5p
		miR-5129-5p

Cell color indicates time point at which microRNAs are DE (p ≤ 0.05). Blue: DE at 0h only. Green: DE at 0 and 8h. Orange: DE at 120h only. No color: Not DE at any time point. Similar superscript indicates microRNAs are from the same family.

& indicates family miR-669a-3p

@ indicates family miR-141-3p

$ indicates family miR-200a-5p

# indicates family miR-200b-3p.

**Table 2:** DE microRNAs common to all 3 brain regions.

	AMY			NAC			PFC		
**microRNA**	**0h**	**8h**	**120h**	**0h**	**8h**	**120h**	**0h**	**8h**	**120h**
**miR-187-3p**	**-1.35**	1.17	-1.17	1.02	-1.00	**-1.13**	**1.61**	-1.16	-1.05
**miR-2137**	**1.26**	1.18	-1.07	**1.29**	1.20	-1.03	**1.32**	1.16	1.05
**miR-7b-3p**	**1.51**	**1.51**	-1.14	**1.30**	1.20	1.05	**1.32**	1.16	-1.05

Three microRNAs were DE in all brain regions (as shown in Fig 2B). Fold changes in bold text indicate significant (p < 0.05) dysregulation at the given time point.

### Temporal patterns of DE microRNAs and associated gene targets

**Fig 4:**
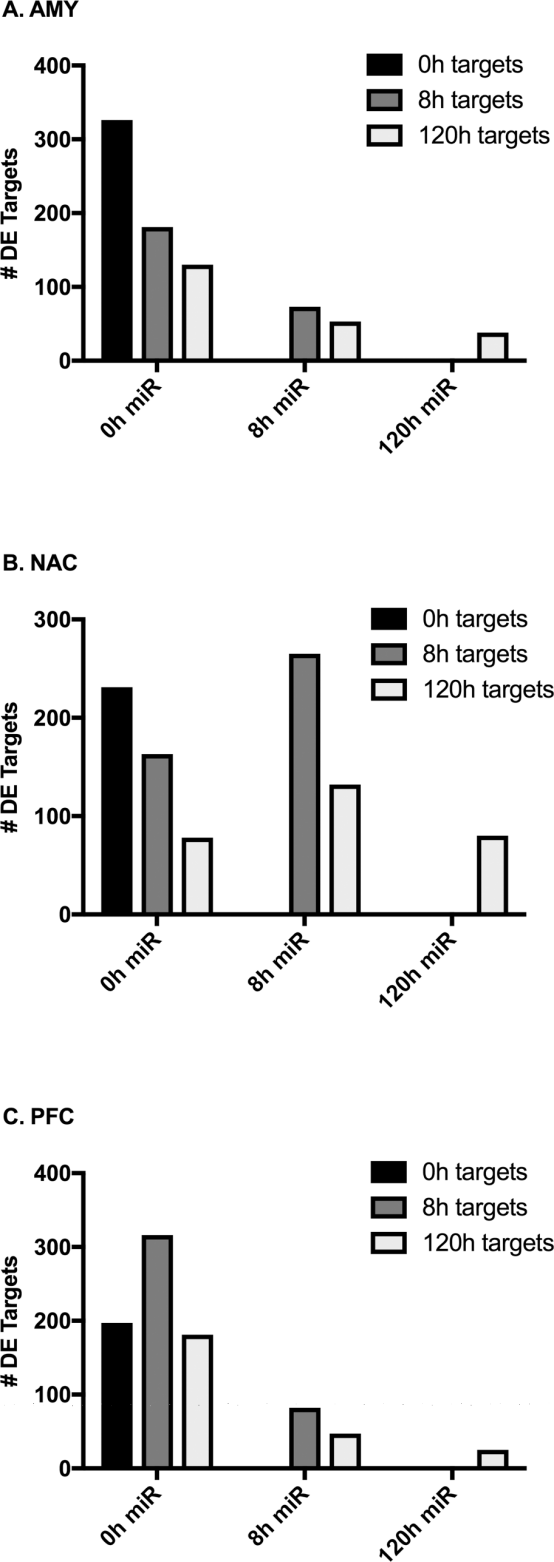
Number of microRNA targets DE at each time point. Bars indicate the number of DE genes that are targets of DE (p < 0.05) microRNAs at the given time point. Gene targets were considered DE at an FDR of 0.05 (0 and 8h targets) or at a nominal value of 0.05 (120h targets).

DE gene targets of the ethanol-responsive microRNAs were identified for each time point. Both experimentally validated and predicted targets (see methods) were utilized. The microRNAs changed at the 0h time point had a number of mRNA targets that were changed at the 0, 8 and 120h time points (Fig 4). In general, microRNAs that were changed at 8 or 120h had fewer targets than those changed at 0h, although NAC showed a number of targets for the microRNAs that were changed at 8h. For each brain region, the proportion of shared 0h microRNA targets was greatest (18–22%) for 0 and 8h (proportion calculated as the number of shared targets divided by the total number of targets in the earlier time point x100; data not shown). Most of the targets of microRNAs were dependent on the time point, however, there were some targets of microRNAs from the 0h point that were also changed at 0, 8 and 120h, or at 0 and 120 h (Table 3). There was little overlap between 8h microRNA targets at 8 and 120h (AMY: 4%, NAC: 7%, PFC: 0%).

**Table 3:** 0h DE microRNA targets dysregulated at multiple time points in each brain region.

Targets dysregulated at 0, 8 and 120h			Targets dysregulated at 0 and 120h only		
AMY	NAC	PFC	AMY	NAC	PFC
Mrpl49	Eml5	Bicd2	Acta2	Arhgap44	Dusp16
Rit1	Mrpl10		As3mt	Desi1	Gng10
Tmem55a	Phldb1		C1orf43	Fabp3	Heatr1
			Ccdc149	Fbxl8	Polr2d
			Diexf	Klc1	Tmem201
			Dusp4	Nt5c2	
			Gss	Pdk3	
			Idh3a	Pip4k2a	
			Nsmf	Rap1b	
			Onecut2	Rhog	
			Prkab1	Spcs3	
			Snn		
			Zeb1		

### Functional annotation of paired DE microRNAs with 120h DE targets

To evaluate the role of microRNAs in the persistent (120h) changes in gene expression, paired DE microRNAs and associated 120h DE targets were assessed with IPA's "Core Analysis" to elucidate the predominant biological functions represented in these data. (See Fig 1 and Methods section "Statistics and Bioinformatics" for details.) Remarkably, hepatotoxicity-related annotations ("liver cancer", "liver carcinoma", "liver hyperplasia/proliferation") were the top toxicological functions found in several NAC and PFC datasets. In the NAC 8hDEmiR/120hDEtargets dataset, "CNS cell death" showed a strong negative z score (-2.3) while in the PFC 0hDEmiR/120hDEtargets dataset, several cognition-related annotations showed strong positive z scores ("formation of cellular protrusions", 2.2; "growth of neurites", 2.2; "cognition", 2.1; "learning", 2.6; "memory", 2.0). "Organization of actin cytoskeleton" and "formation of lymphocytes" were the most significant annotations in AMY, occurring in the 0hDEmiR/120hDEtargets dataset with z scores of 1.98 and 2.0, respectively.

### Hierarchical clustering of DE microRNAs

**Fig 5:**
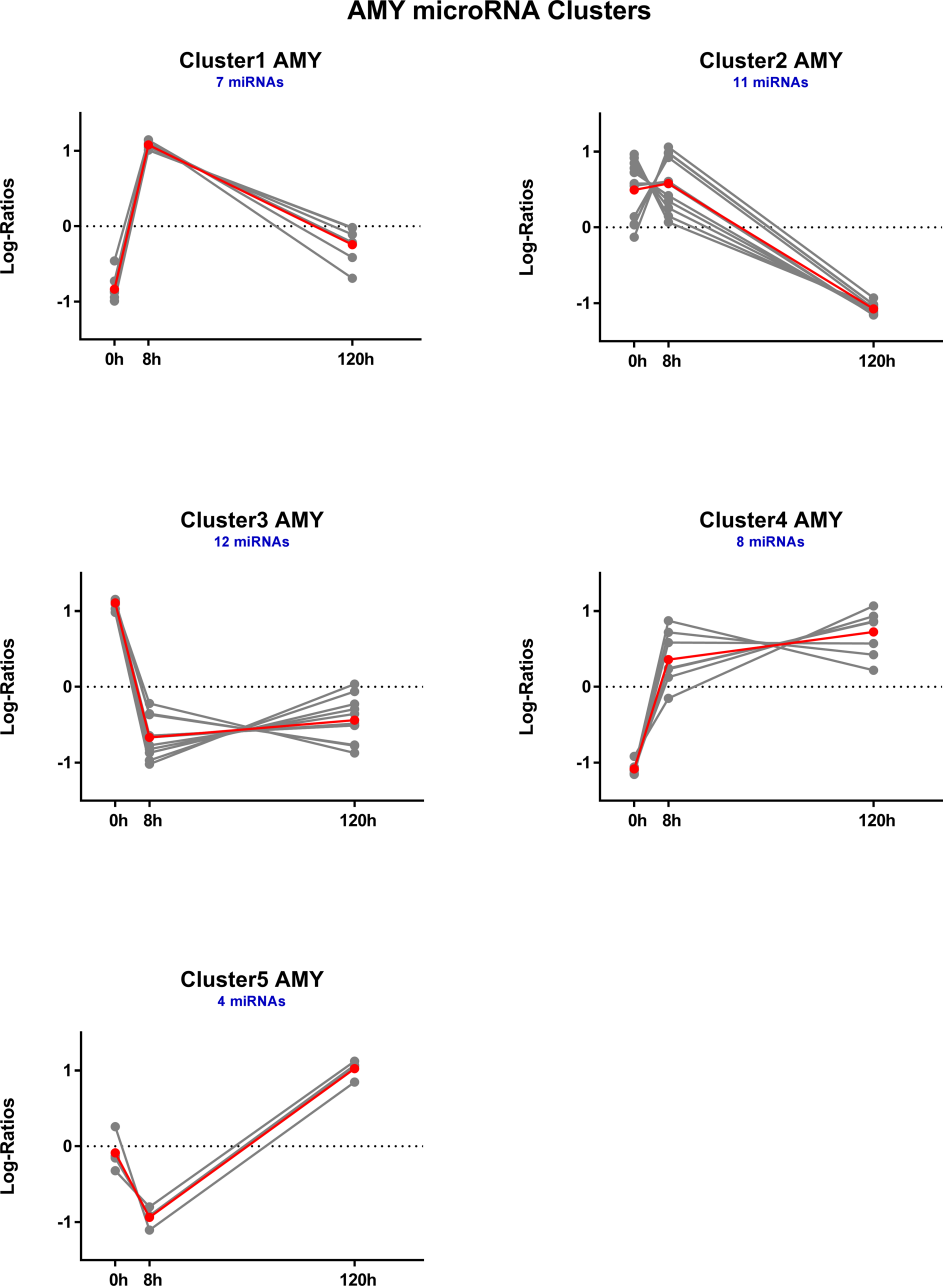
Temporal profiles of hierarchically clustered, DE (p < 0.05) microRNAs in amygdala. Profiles are based on hierarchical clustering of centered and scaled expression log ratios of microRNAs DE in at least one time point. Average expression is plotted in red, and each individual microRNA is plotted in gray. Blue text indicates the total number of microRNAs in each cluster.

**Fig 6:**
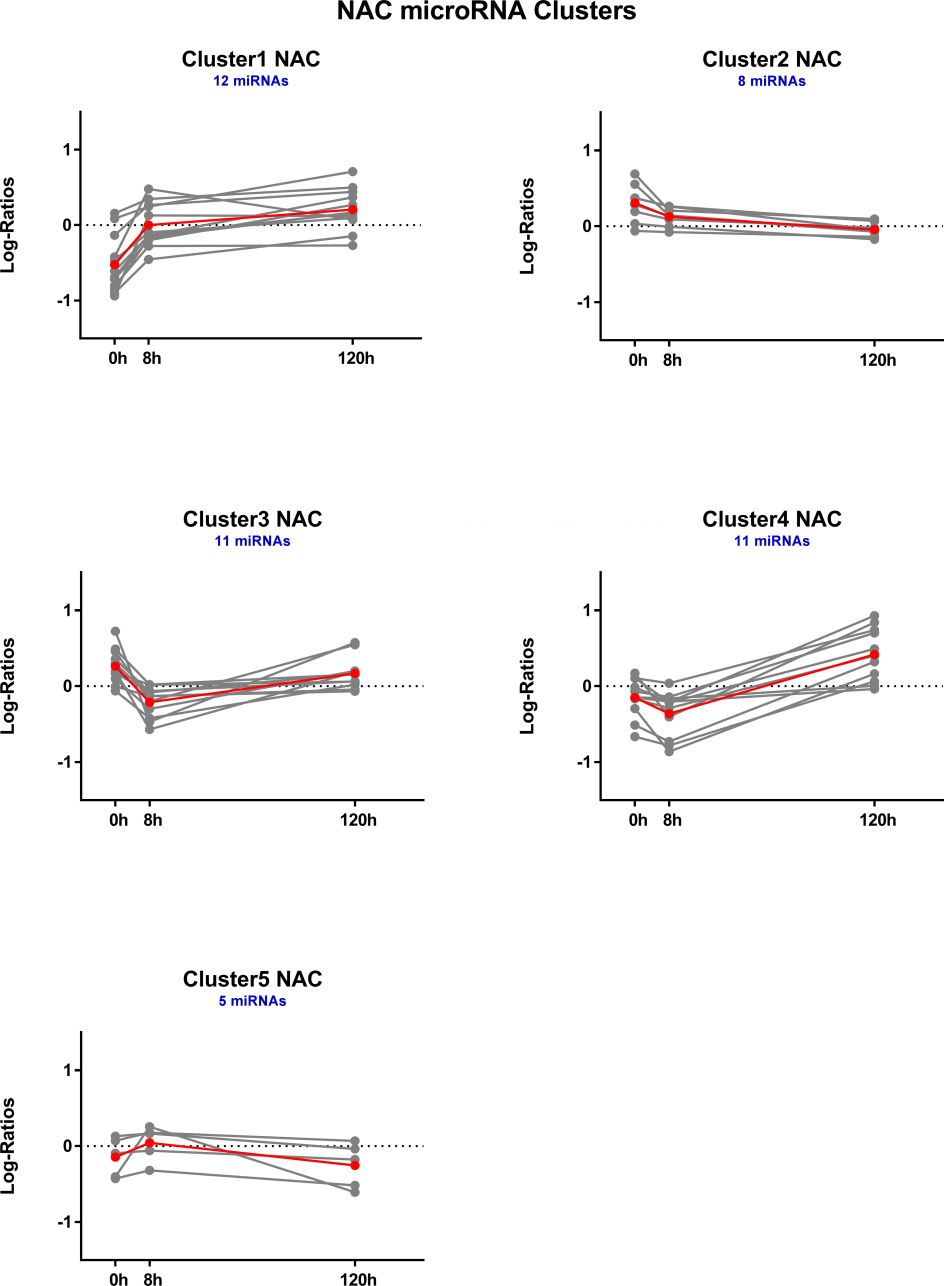
Temporal profiles of hierarchically clustered, DE (p < 0.05) microRNAs in nucleus accumbens. Profiles are based on hierarchical clustering of centered and scaled expression log ratios of microRNAs DE in at least one time point. Average expression is plotted in red, and each individual microRNA is plotted in gray. Blue text indicates the total number of microRNAs in each cluster.

**Fig 7:**
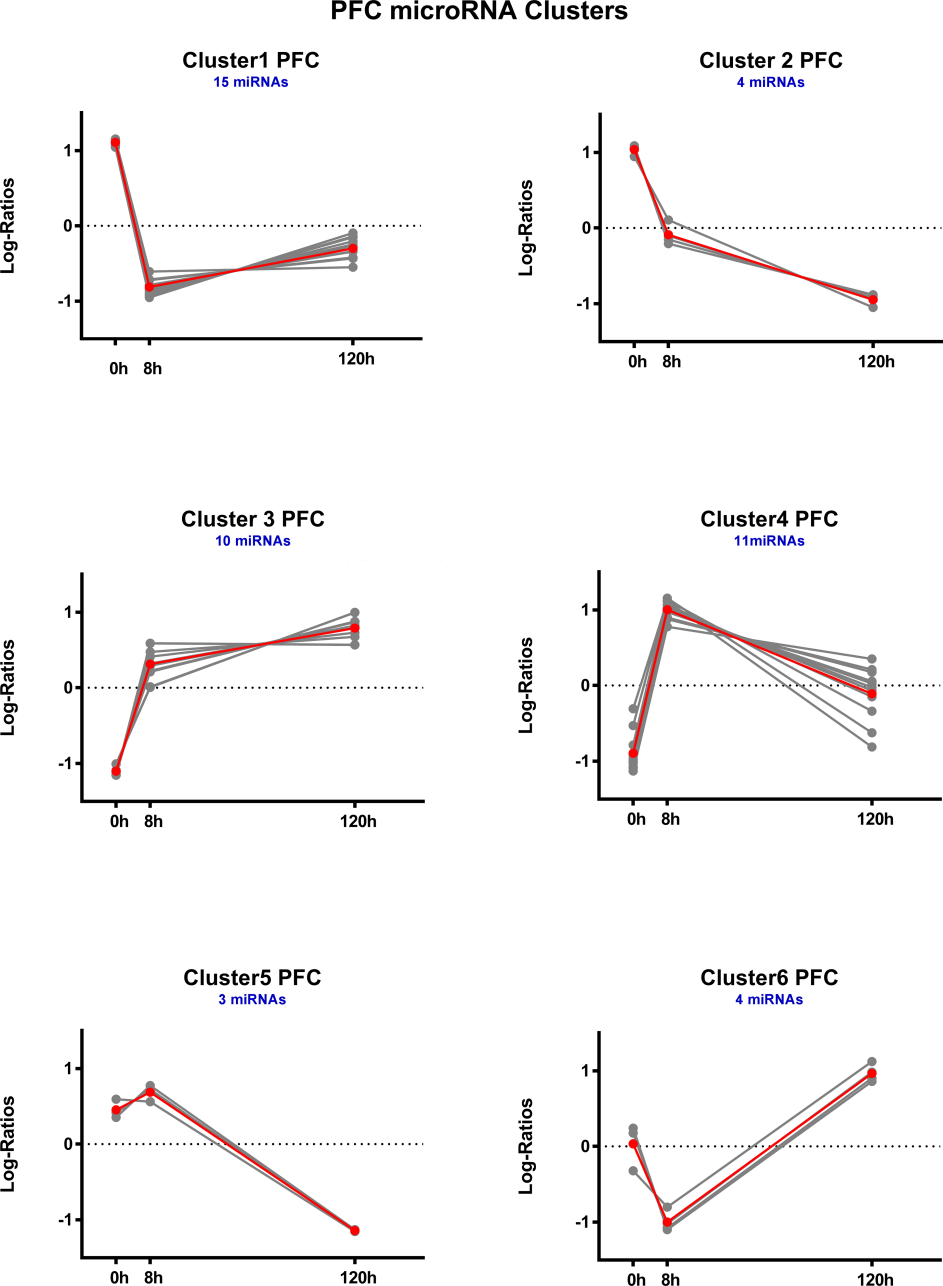
Temporal profiles of hierarchically clustered, DE (p < 0.05) microRNAs in prefrontal cortex. Profiles are based on hierarchical clustering of centered and scaled expression log ratios of microRNAs DE in at least one time point. Average expression is plotted in red, and each individual microRNA is plotted in gray. Blue text indicates the total number of microRNAs in each cluster.

For each brain region, DE microRNAs were clustered based on expression log ratios. This resulted in 5 distinct temporal clusters each for AMY (Fig 5) and NAC (Fig 6) and 6 clusters for PFC (Fig 7). See also S3 Table.

### Time point-based network analysis

As part of the "Core Analysis", IPA uses "network eligible molecules" (those molecules in the dataset that interact with other molecules in the IPA knowledgebase) as "seeds" to generate networks with a high degree of connectivity relative to all molecules in the knowledgebase. These networks provide insight to molecule connections and relationships that may not be detected by standard functional annotation methods. Using this approach, we compared the top two networks derived from each dataset containing DE microRNAs and their associated DE 120h targets (Table 4, S2 Fig). All datasets, except PFC 8hDEmiR/120hDEtargets, produced top networks in which *ERK1/2* (mouse equivalent *Mapk3*, *Mapk1*) was a highly-connected member. These same networks contained other highly connected molecules, including Bcl2 (in AMY networks) and *Srf* (in PFC networks). In NAC and PFC, datasets utilizing targets from 0 and 8h microRNAs produced *NF-kB*-centered networks, while the same datasets in AMY generated networks sharing *Smad3*.

**Table 4:** Top IPA networks sharing common hub genes.

Dataset	Network attributes	AMY		NAC		PFC	
**0hDEmiR/ 120hDEtargets**	Rank	1	2	2	2	1	2
	Score	39	32	41	41	41	36
	# Focus Molecules/ # Dataset Molecules	21/47	21/47	21/87	21/87	23/197	21/197
**8hDEmiR/ 120hDEtargets**	Rank	1	1	1	2		1
	Score	43	43	45	39		41
	# Focus Molecules/ # Dataset Molecules	19/58	19/58	23/141	21/141		18/50
**120hDEmiR/ 120hDEtargets**	Rank		1	1		1	
	Score		40	36		21	
	# Focus Molecules/ # Dataset Molecules		17/41	18/87		11/28	
	Shared Hub Genes	Smad3	Bcl2, Erk1/2	Erk1/2	NF-kB	Srf, Erk1/2	NF-kB

Paired datasets of 0, 8 or 120h DE microRNAs and their 120h DE targets were used in conjunction with IPA Knowledgebase molecules to generate highly connected networks. Each network contained 35 molecules. Statistical fit between each network and its source dataset was evaluated with a right tailed Fisher's exact test. Rank indicates relative statistical significance among all derived networks with the lowest rank (1) representing the most significant network. Network score is the -log_10_ of Fisher's exact p value. Focus molecules are network molecules originating from the dataset. Hub genes were identified based on high connectivity in multiple networks derived from different datasets.

**Fig 8:**
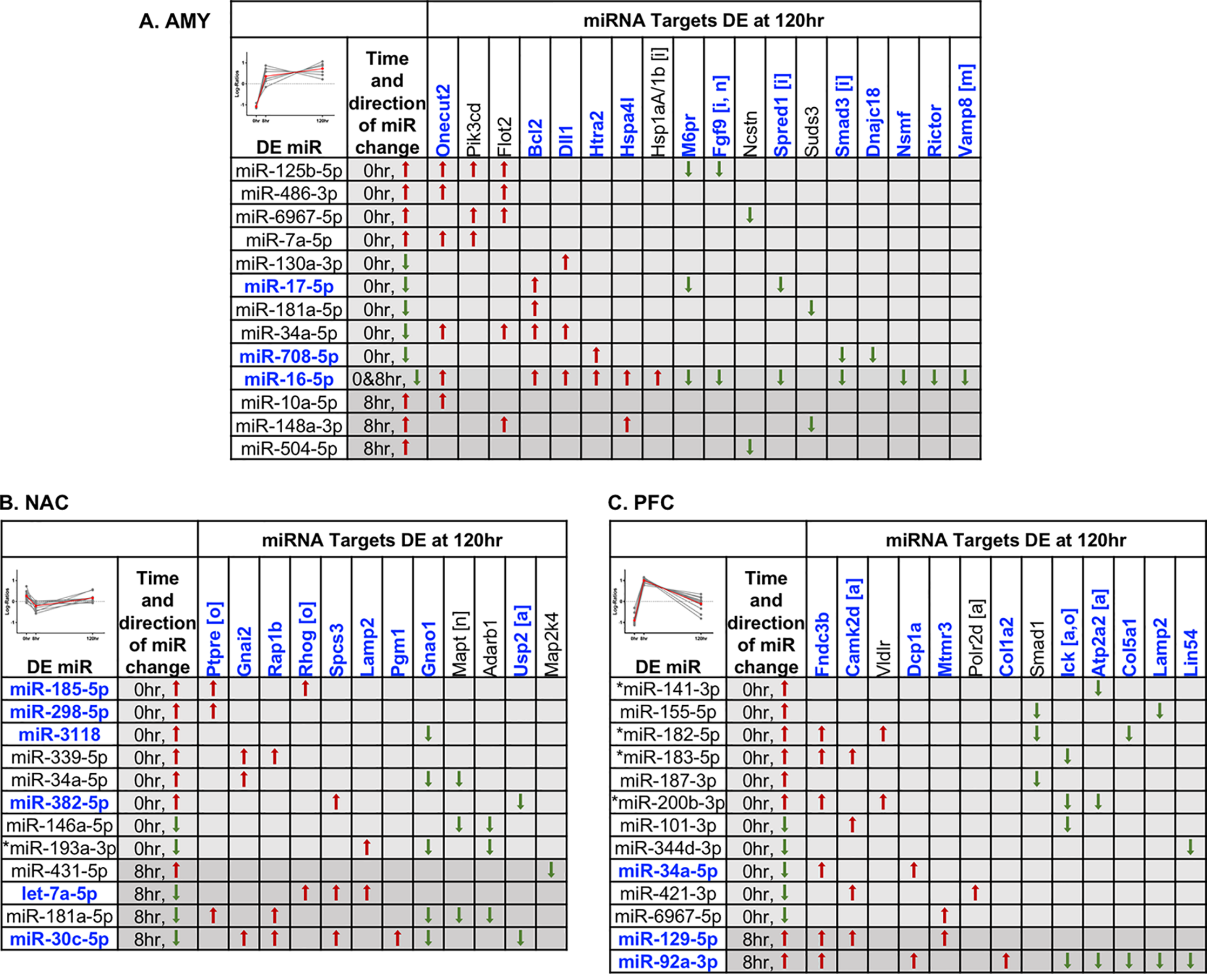
Critical network genes (mRNAs) DE at 120h and their potential microRNA regulators DE at 0 or 8h. Only genes with an associated microRNA are listed. Critical network genes were dysregulated at 120hr and present in at least one of the top three IPA networks derived from the time-point based network analysis. Blue text indicates that RNAs were also identified in the cluster sharing the greatest overlap with the list of critical network genes: AMY—cluster 4; NAC—cluster 3; PFC: cluster 4. Inset shows temporal expression pattern for the designated cluster. See S5 Fig for a full version of the inset figures. Red upward pointing arrow indicates up-regulation; Green downward pointing arrow indicates down-regulation. Bracketed letters denote membership in cell-type specific gene lists (See [26]) enriched in the analyzed datasets [a = astrocyte, i = immune related, m = microglia, n = neuron, o = oligodendrocyte.] (Cell specific gene lists were pre-loaded into IPA and automatically scored against all datasets submitted to Core Analysis. Significant enrichment was determined by p value < 0.05 using Fisher’s exact test.) A single asterisk (*) indicates the microRNA was uniquely detected in the indicated brain-region.

We further employed a process of network merging to uncover the most important relationships between 120h DE genes and their associated microRNAs dysregulated during 0 and 8h time points. For each brain region, the top 3 networks obtained from the "0hDEmiR/120hDEtargets" and "8hDEmiR/120hDEtargets" datasets were combined, expanding component gene families or complexes to include all members present in the dataset (S3 Fig). Genes DE at 120h and common to these 2 merged networks are given in Table 5. Using the time point-based microRNA-mRNA relationships, we identified potential dysregulated microRNA regulators of these critical network genes (Fig 8).

**Table 5:** Critical network genes.

AMY			NAC			PFC		
Gene	FC	pval	Gene	FC	pval	Gene	FC	pval
***Bcl2***	1.04	9.2E-03	**Adarb1**	-1.03	7.6E-03	**Atp2a2**	-1.07	3.7E-02
**Dll1**	1.02	2.0E-02	**Gnai2**	1.07	3.0E-02	**Camk2d**	1.04	1.3E-02
**Dnajc18**	-1.04	5.4E-03	**Gnao1**	-1.08	3.6E-02	**Col1a2**	1.04	1.8E-03
**Dnajc28**	-1.03	3.7E-02	**Lamp2**	1.07	2.8E-03	**Col5a1**	-1.05	3.1E-02
**Fgf9**	-1.06	7.5E-05	**Map2k4**	-1.05	4.6E-02	**Dcp1a**	1.04	3.5E-02
**Flot2**	1.04	8.6E-03	***Mapt***	-1.04	4.1E-02	***Fndc3b***	1.04	4.8E-02
**Hsp90ab1**	1.09	1.4E-02	**Pgm1**	1.08	1.1E-02	**Ick**	-1.03	4.2E-02
**Hspa1a/1b**	1.04	2.2E-02	Ppp2r1a	-1.06	2.5E-02	**Lamp2**	-1.06	3.6E-02
**Hspa4l**	1.03	4.9E-02	**Ptrpe**	1.08	1.4E-02	**Lin54**	-1.05	1.5E-02
**Htra2**	1.05	1.2E-02	**Rap1b**	1.06	2.1E-02	**Mtmr3**	1.04	5.6E-03
**M6pr**	-1.10	2.3E-03	Rela	1.05	2.6E-02	**Polr2d**	1.04	1.7E-02
**Mapk6**	1.04	2.9E-02	**Rhog**	1.06	1.9E-02	**Smad1**	-1.07	4.8E-03
**Ncstn**	-1.05	1.2E-02	**Spcs3**	1.04	3.2E-02	**Vldlr**	1.07	2.4E-02
**Nsmf**	-1.09	4.2E-02	**Usp2**	-1.04	4.8E-02			
**Onecut2**	1.04	1.4E-02						
**Pik3cd**	1.03	5.0E-02						
**Rictor**	-1.08	1.3E-02						
**Smad3**	-1.11	9.0E-03						
**Spred1**	-1.05	4.1E-02						
**Suds3**	-1.06	1.2E-02						
**Vamp8**	-1.04	3.4E-02						

Genes dysregulated at 120h and present in at least one of the top three IPA networks derived from miRNAs dysregulated at 0 and 8h. Bold text indicates 120h dysregulated targets of miRNAs differentially expressed at 0 or 8h. Plain text indicates genes dysregulated at 120h that are not targets of 0 or 8h DE miRNAs. Hub genes derived from hierarchical clustering analyses are denoted with italics.

#### Cluster-based network analysis

Networks were created from each cluster of microRNAs and their associated targets DE at any time point, emphasizing those targets dysregulated at 120h (S4 Fig). Notably, genes identified as hubs in the cluster-based networks (Table 6) were also identified as hubs in the network analysis of paired microRNAs and 120h DE targets. For example, *Bcl2* showed high connectivity in AMY networks created from both approaches; similarly, for *Srf* in PFC networks. Additionally, hub genes from the cluster-based networks were also essential members of the combined networks described above. These included *Bcl2* in AMY, *Mapt* in NAC and *Fndc3b* in PFC (Tables 5 and 6). In general, cluster-based networks exhibited varying degrees of overlap with critical network genes identified in the time point-based analysis (Table 5). The greatest overlap occurred between Table 5 genes and AMY cluster 4 (13/21 genes), NAC cluster 3 (9/14 genes) and PFC cluster 4 (10/13 genes) (S5 Fig). MicroRNAs and mRNAs identified by both cluster- and time point-based approaches are highlighted in Fig 8.

**Table 6:** Hub genes identified in the cluster-based networks and their associated microRNAs.

microRNA						mRNA				
Cluster	MicroRNA connected to hub gene	Change	Expression p-value	Time DE	MicroRNAs dysregulated in PFC of human alcoholics and/or alcohol-dependent rats	Hub gene	Change	Expression p-value	Time DE	Species and mRNA reference link
**AMY Cluster1**	**miR-181a-5p**	**⬇**	2.71E-02	0h	-	***Bcl2***	**⬆**	9.16E-03	120h	Rat (Ref)
	**miR-34a-5p**	**⬇**	1.93E-02	0h	-					
**AMY Cluster2**	**miR-125b-5p**	**⬆**	4.44E-02	0h	rno-miR-351_st	***Bcl2***	**Same as above**			
**AMY Cluster3**	**miR-2861**	**⬆**	2.73E-02	0h	-	**Map2k7**	**⬇**	7.64E-03	120h	Human (Ref)
	**miR-3960**	**⬆**	4.91E-02	0h	-					
	**miR-486-3p**	**⬆**	4.15E-02	0h	-					
**AMY Cluster4**	**miR-16-5p**	**⬇**	1.58E-02	0h	rno-miR-195_st/hsa-miR-15b-5p	***Bcl2***	**Same as above**			
	**miR-17-5p**	**⬇**	3.25E-02	0h	rno-miR-93_st					
	^**+**^**miR-503-5p**	**-**	-	-	-					
**AMY Cluster5**	^**+**^**mir-322**	**-**	-	-	-	***Bcl2***	**Same as above**			
	**miR-16-5p**	**⬇**	1.98E-02	8h	rno-miR-195_st/hsa-miR-15b-5p					
**NAC Cluster1**	**miR-146a-5p**	**⬇**	4.05E-02	0h	hsa-miR-146a-5p	***Mapt***	**⬇**	4.05E-02	120h	Rat (Ref)
**NAC Cluster2**	**miR-34a-5p**	**⬆**	4.22E-02	0h	hsa-miR-34c-5p	**Hnf4a**	**⬇**	4.58E-02	120h	Rat (Ref)
**NAC Cluster3**	**miR-3118**	**⬆**	4.44E-02	0h	-	**Egfr**	**⬆**	2.91E-03	8h	Drosophila (Ref)
**NAC Cluster4**	**miR-7a-5p**	**⬆**	1.63E-02	120h	rno-miR-7a_st/hsa-miR-7-5p	**Egfr**	**Same as above**			
**NAC Cluster5**	**miR-129-1-3p**	**⬆**	2.64E-02	8h	rno-miR-129-star_st	**Bag3**	**⬆**	2.76E-02	120h	Human (Ref)
**PFC Cluster1**	**miR-141-3p**	**⬆**	2.79E-02	0h	-	**Ctnnb1**	**⬇**	4.56E-02	120h	Avian (Ref)
**PFC Cluster2**	**miR-141-3p**	**⬆**	4.19E-02	0h	-	**Ctnnb1**	**Same as above**			
	**miR-155-5p**	**⬆**	2.22E-03	0h	-					
**PFC Cluster3**	**miR-101-3p**	**⬇**	1.81E-02	0h	rno-miR-101b_st/hsa-miR-101-3p	**Srf**	**⬆**	9.14E-04	120h	Ferrets (Ref)
	**miR-329-3p**	**⬇**	4.00E-02	0h	-					
	**miR-335-5p**	**⬆**	3.98E-02	120h	rno-miR-335_st					
**PFC Cluster4**	**miR-129-5p**	**⬆**	4.38E-02	8h	-	***Fndc3b***	**⬆**	4.77E-02	120h	Human (Ref)
	**miR-188-5p**	**⬇**	1.80E-02	120h	-	**Rbfox2**	**⬆**	2.54E-03	8h	Human (Ref)
	**miR-34a-5p**	**⬇**	3.06E-02	0h	hsa-miR-34c-5p	**Sh3kbp1**	**⬆**	3.08E-02	120h	Mouse (Ref)
**PFC Cluster5**	**miR-92a-3p**	**⬆**	3.12E-02	8h	rno-miR-92b_st/hsa-miR-92a-3p	**Sdhd**	**⬇**	1.91E-02	120h	Human (Ref)
**PFC Cluster6**	**miR-3093-3p**	**⬆**	4.58E-02	8h	-	**Srf**	**Same as above**			
	**miR-486-5p**	**⬆**	9.37E-03	120h	-					

Hub genes, affiliated microRNAs and expression data for both (direction and time of change, nominal p value) are provided for each cluster. MicroRNAs preceded by a "+" superscript were not DE in our dataset but were added and connected by the IPA algorithm. Italics denote critical network genes (see Table 5). The last column of microRNA data lists several hub gene-connected microRNAs that were also dysregulated in prefrontal cortex of human alcoholics [10] and/or alcohol-dependent rats [9]. These microRNAs are provided as probeset IDs or microRNA names, depending on their presentation in the reference. The last column of mRNA data reports publications that demonstrate a link between ethanol exposure and expression changes for the given hub gene in each miRNA cluster. References are provided as a hyperlink and are prefixed with the abbreviation for the studied species.

## Discussion

Gene expression profiling studies demonstrate clearly that alcohol consumption changes brain region-specific transcriptional profiles in human alcoholics and animal models of voluntary consumption [40]. We recently reported time-dependent changes in mRNA expression in mice exposed to chronic intermittent ethanol (CIE) exposure (a model of alcohol dependence). It is well established that miRNAs can alter the expression of many target genes and a number of studies have shown that miRNA expression is altered in response to alcohol abuse in humans [10–13] and in animal models [7, 9, 14–17]. Rodent drinking models have been important in identifying alcohol-responsive miRNAs and their functional relevance based on responses to expression manipulation. Alcohol-induced changes in microRNAs are associated with cellular tolerance to alcohol [41], antianxiety effects [42], cellular reward mechanisms [20], regulation of alcohol consumption and preference [16, 17, 20, 21], episodes of binge drinking [7, 14, 43], dependence/withdrawal [9, 15, 17] and alcohol-induced conditioned-place preference [21]. However, these studies report expression changes that are occurring at a single point in time after alcohol treatment and thus, do not capture dynamic transcriptional regulation. The temporal relationship between alcohol-responsive miRNAs and mRNAs resulting from alcohol challenge has not been investigated directly. The goal of this study was to combine new microRNA data with previously identified mRNA [26] changes in brain regions of mice subjected to CIE vapor, a paradigm known to escalate voluntary ethanol consumption in rodents. MicroRNA expression changes were measured in three brain regions (AMY, NAC, PFC) at three time points (0-, 8- and 120h) following repeated exposures to ethanol vapor. These three time points represent distinct actions of ethanol as they correspond to intoxication, withdrawal and protracted abstinence, respectively. These very different actions of alcohol would be expected to have brain-region and gene network specific actions on the transcriptome as we found in our earlier study (24). Because we propose that at least some of the transcriptome changes are related to changes in microRNAs, we would also expect that the DE microRNAs would vary considerably between time points and brain regions.

Indeed, DE probesets were distinct for the brain regions (see Fig 1) and within each brain region overlap of DE probesets across time was limited (see S1 Fig). Within each brain region the mRNA response to CIE also produced a fairly unique profile of DE genes at each time point. This emphasizes the unique time- and brain-region specific alterations that occur following CIE vapor. Notably, the temporal patterns of microRNA and mRNA expression patterns differed (see Fig 2). All brain regions exhibited a decrease in DE microRNAs from 0 to 8h and a concomitant reduction of DE mRNAs between 8 and 120h, likely reflecting the time required for the microRNAs to exert their effects on gene targets. These findings are consistent with those identified in previous studies in mouse [7] and human alcoholics [10] which demonstrate over-represented directional changes based on time of alcohol exposure. In contrast to AMY and NAC, the number of DE microRNAs at 120h continued to decline in the PFC, perhaps indicating that microRNA regulation in the PFC is under greater homeostatic regulatory control given the complexity of the transcriptome networks responding to alcohol challenge [7, 44]. In PFC, several DE microRNAs are members of the same family, especially at the 0h time point. (S2 Table). The greater redundancy of DE microRNA family members in PFC compared with AMY and NAC could suggest a higher degree of transcriptional fine tuning or regulatory prioritization in this brain region. In addition, temporal patterns of DE microRNA gene targets also varied significantly among brain regions. AMY and PFC microRNAs were most responsive to the direct effects of ethanol (0h), whereas NAC displayed a greater response to ethanol withdrawal (8h). This is supported by the finding that the greatest number of DE gene targets in AMY and PFC were associated with microRNAs DE at 0h while the greatest number of DE gene targets in NAC were associated with microRNAs DE at 8h (Fig 4).

We reasoned that integrating differential expression profiles from both microRNAs and mRNAs would provide greater insight into the perturbed gene networks associated with withdrawal/protracted withdrawal. Our microRNA-target association procedure allowed for both perfect and imperfect sequence matching between microRNA-mRNA pairs. This undoubtedly influenced our results since a microRNA’s function is determined in part by the extent of sequence complementarity with its target. In general, a perfect sequence match between microRNA and target is believed to cause mRNA degradation while an imperfect match is believed to result in translational repression [45]. Thus, utilizing only perfect-match microRNA-mRNA pairs would have omitted non-degraded mRNA targets from our analysis, potentially resulting in more precise associations. However, the relationship between microRNA-mRNA sequence complementarity and microRNA function is not absolute; and limiting our analysis exclusively to perfectly matched pairs would likely result in a significant loss of information. Thus, we opted to include both perfect and imperfect sequence matches, relying on dual analyses to ensure reliable results. Two different strategies were used to reveal relationships between DE microRNAs and their targets: discrete time-based analysis and a hierarchical cluster-based analysis.

Interestingly, divergent analytical approaches identified a number of common genes that were highly connected within networks (connectivity and hubs). These analyses have identified a number of neuroimmune- and cell death/survival-related pathways including *ERK1/2* (mouse equivalent *Mapk3*, *Mapk1*; time-point based paring only), *Bcl2* (in amygdala networks) and *Srf* (in prefrontal cortex networks). These identified hub genes demonstrated robust microRNA-mRNA network alterations in response to alcohol exposure. In addition, temporal analyses identified *NF-kB*- and *Smad3*-centered networks in NAC and PFC. Mitogen-activated protein kinases (*Mapk3* and *Mapk1*) are critical components of signal transduction pathways that are involved in cell growth, adhesion, survival and differentiation [46] as well as myelination [47]. *Rit1*, a microRNA target dysregulated at multiple time points in each brain region, was also identified in the current study (see Table 3). This gene is dependent upon MAP kinase signaling pathways which are involved in neuritogenesis [48]. These biological functions are associated with regulation of transcription, translation, and cytoskeletal rearrangements [49]. In addition, these pathways are activated by a variety of signals including cytokines and heterotrimeric G protein coupled receptors [50]. Both *Mapk3* and *Mapk1* are related to alcohol actions in mouse [51, 52] and human [53] brain. They have also been implicated in addiction to other drugs of abuse, such as cocaine [54]. *Bcl2* (B-cell lymphoma 2) family proteins regulate cell death by either pro- or anti-apoptotic mechanisms and *Bcl2* is considered a "prosurvival" protein that is regulated by the *Erk1/2* signaling pathway [55]. It was reported recently that ethanol exposure results in pre-mRNA mis-splicing of myeloid cell leukemia-1 (Mcl-1) which is an anti-apoptotic member of the Bcl-2 family of proteins, suggesting a potential role for *Bcl2* in ethanol-mediated neuronal cell death [56]. The regulation of *Bcl*-related family members is clearly important to understand in the alcohol field given that alcohol in a variety of settings is known to be involved in cell death [57]. In addition, *Bcl2* is upregulated in alcohol preferring mice [51] and protects against ethanol neurotoxicity in neonatal mouse cerebellum [58].

*NF-kB*- and *Smad3* networks were identified as highly connected in NAC. *NF-kB* is a ubiquitously expressed transcription factor family that controls the transcription of hundreds of genes that are involved in many processes, including inflammation, immunity, cell proliferation, and cell death [59, 60] and is known to be an important regulator of neuroinflammation [61]. A number of studies have identified genes that are related to inflammatory/immune responses and mediate their effects through *NF-kB* [40]. *Smad3* (SMAD family member 3) mediates signaling from transforming growth factor beta (*TGF-β*) that is a regulator of cell proliferation, differentiation and death [62]. *Smad3* knockout mice have impaired immune function, suggesting *SMAD* signaling is involved in regulating the immune response [63, 64]. Recently, alcohol-induced microglial changes were found to be over-represented in genes attributed to *TGF-β/Smad3* receptor signaling and inflammatory response [65]. Given that *TGF β* is a cytokine expressed in brain that is capable of controlling microglial activation, this signaling pathway may have the potential to regulate alcohol consumption. MicroRNAs that targeted these hubs included miR-34a-5p, miR-17-5p, miR-181a-5p, miR-16-5p for *Bcl2* in the AMY (except for miR-16-5p, these microRNAs are involved with immune response [66]), and miR-335-5p for *Bcl2* in the PFC (identified in the time-point and cluster based pairings). In NAC, miR-34a-5p and miR-146a-5p are potentially important regulators of Erk1/2 and *NF-kB* signaling pathways (the time-point based pairings only), respectively.

These results emphasize the importance of investigating the complex temporal relationships that exist between miRNA and gene expression changes in response to alcohol challenge. Many studies have focused on "snapshots" in time to establish the relationship between expression changes of miRNA and mRNA; however, this temporal relationship is complex and difficult to study. The current studies are unique because they address critical questions about the temporal relationship between miRNA regulation and mRNA function. These results demonstrate that alcohol exposure results in complex temporal changes in microRNA-mRNA gene network structure and that manipulation of microRNAs may rescue the aberrant synaptic plasticity associated with alcohol consumption.

## Supporting information

S1 Fig Overlap of DE probesets within each brain region as a function of time. Panel A = AMY (amygdala), Panel B = NAC (nucleus accumbens), Panel C = PFC (prefrontal cortex).
(PPTX)

S2 Fig Top time-point based networks sharing hub genes. Gene networks and associated microRNA and mRNA expression data for networks described in Table 4. Hub genes are highlighted in blue. Molecules are colored to indicate direction of dysregulation (treated vs control) at the time point listed in the table. Red = upregulated, green = downregulated. Datasets follow the naming convention outlined in S1 Table and are further prefixed by the appropriate brain region abbreviation. Networks are numbered according to the rank assigned by IPA, with the lowest rank representing the most significant network. Data are exported from IPA and thus utilize human gene nomenclature.
(XLSX)

S3 Fig Temporal miRNA regulation of target genes. To investigate the potential time-dependent relationship of miRNA gene regulation, focused datasets were created from differentially expressed miRNAs (0 and 8 h only) and their differentially expressed target mRNAs (120h). For each of these miRNA-mRNA datasets, genes from the top three IPA-derived networks were merged and genes common to the merged networks were identified. This enabled us to focus on a key list of genes whose expression is modulated by miRNAs during withdrawal and remains dysregulated during protracted withdrawal, a period of time associated with drug relapse.
(PPTX)

S4 Fig Cluster-based microRNA-mRNA networks constructed in IPA. Networks were created from each cluster of microRNAs and their associated targets DE at any time point, emphasizing those targets dysregulated at 120h. Font color indicates time of DE (nominal p < 0.05). Maroon = DE at 0h, green = DE at 8h, blue = DE at 120h, gray = not DE at any time point. Solid and dashed lines indicate direct and indirect relationships, respectively. Hub genes are highlighted with a light blue box; their relationships to other molecules are indicated by a pink line. Molecules are colored to indicate direction of dysregulation (treated vs control) at the time point listed in the table. Red = upregulated, green = downregulated. Data are exported from IPA and thus utilize human gene nomenclature.
(XLSX)

S5 Fig AMY microRNA cluster exhibiting the greatest overlap with critical network genes identified in the time-point based network analysis. Average expression is plotted in red, and individual microRNAs are plotted in gray. Inset table provides expression data for microRNAs in the cluster. Time ("Time DE") and direction ("Change") of change are given for each microRNA (⬆ = up-regulation; ⬇ = down-regulation). The number and direction of 120h DE targets are given for each microRNA.
(TIF)

S6 Fig NAC microRNA cluster exhibiting the greatest overlap with critical network genes identified in the time-point based network analysis. Average expression is plotted in red, and individual microRNAs are plotted in gray. Inset table provides expression data for microRNAs in the cluster. Time ("Time DE") and direction ("Change") of change are given for each microRNA (⬆ = up-regulation; ⬇ = down-regulation). The number and direction of 120h DE targets are given for each microRNA.
(TIF)

S7 Fig PFC microRNA cluster exhibiting the greatest overlap with critical network genes identified in the time-point based network analysis. Average expression is plotted in red, and individual microRNAs are plotted in gray. Inset table provides expression data for microRNAs in the cluster. Time ("Time DE") and direction ("Change") of change are given for each microRNA (⬆ = up-regulation; ⬇ = down-regulation). The number and direction of 120h DE targets are given for each microRNA.
(TIF)

S1 Table Names of paired datasets used for discrete temporal analysis. ND = not done.
(DOCX)

S2 Table Multiple microRNAs differentially expressed at 0h in PFC are from the same family. MicroRNA families are derived from IPA and include all microRNAs with the same seed sequence. Colored cells identify microRNA families with multiple members dysregulated at 0h in PFC.
(DOCX)

S3 Table Expression data and cluster membership for DE (p < 0.05) microRNAs in each brain region. Centered and scaled expression log ratios of DE microRNAs were hierarchically clustered using the R package clValid [38]. The table lists microRNA probe IDs and associated mirBase (v21) and IPA names. Clusters are identified by a unique number in each brain region. Expression data are the original (unscaled/uncentered) values. logFC = log fold change of treated vs control.
(XLSX)

S4 Table DE data for all probesets detected above background. The table lists microRNA probe IDs and associated mirBase (v21) names. Fold changes (treated vs. control), p values and false discovery rates (FDR) were determined using empirical Bayes moderated t-statistics from the Bioconductor package limma.
(XLSX)
