# Supplementary figures and images for: Long-term ethanol exposure: Temporal pattern of microRNA expression and associated mRNA gene networks in mouse brain
Source: PLoS One. 2018 Jan 9;13(1):e0190841. doi: 10.1371/journal.pone.0190841 (PMC5760035; doi:10.1371/journal.pone.0190841)

## Slide 1
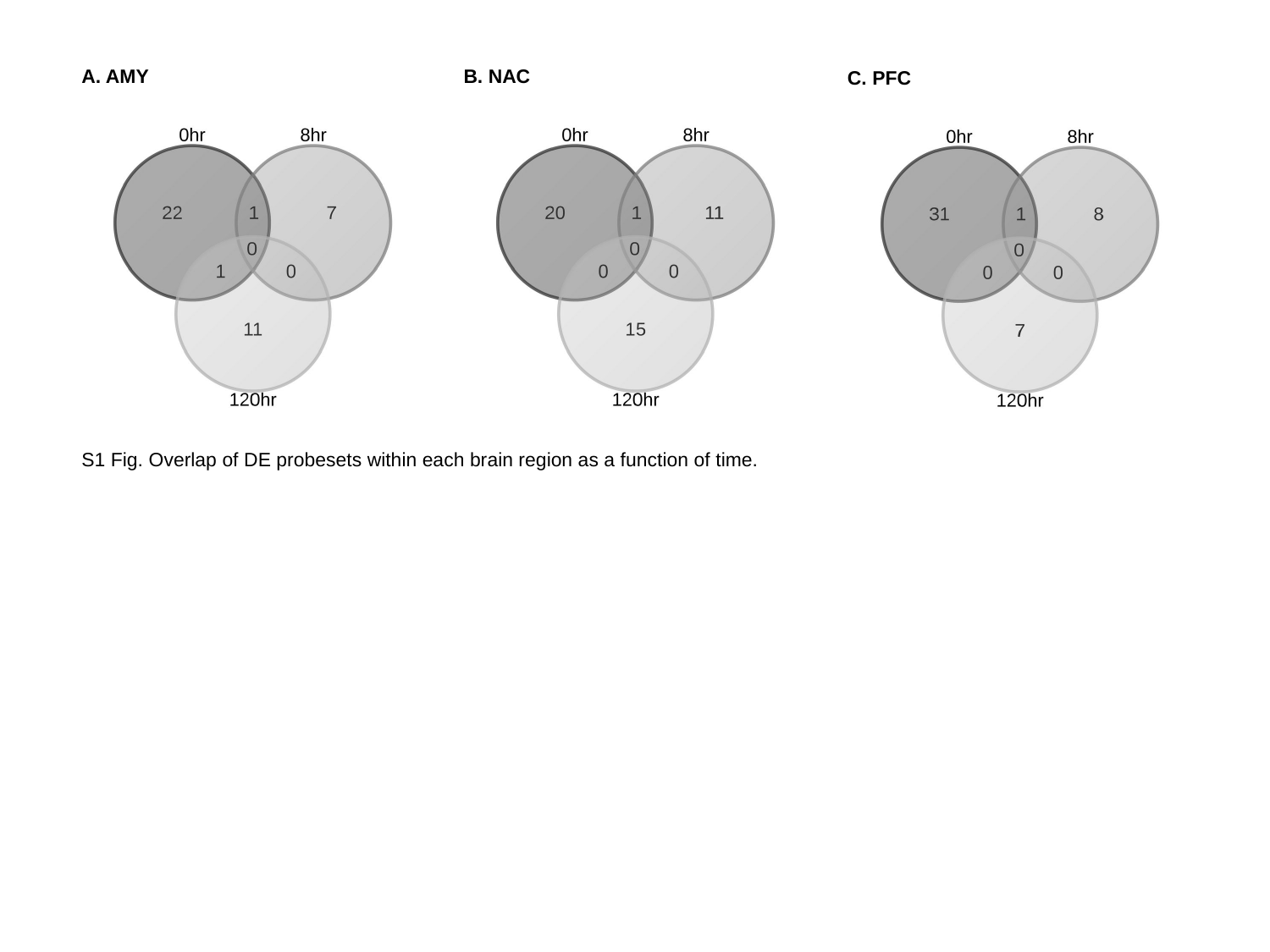

A. AMY
B. NAC
C. PFC
# S1 Fig. Overlap of DE probesets within each brain region as a function of time.

Supplement: S1 Fig — Panel A = AMY (amygdala), Panel B = NAC (nucleus accumbens), Panel C = PFC (prefrontal cortex). (PPTX) [file pone.0190841.s001.pptx]

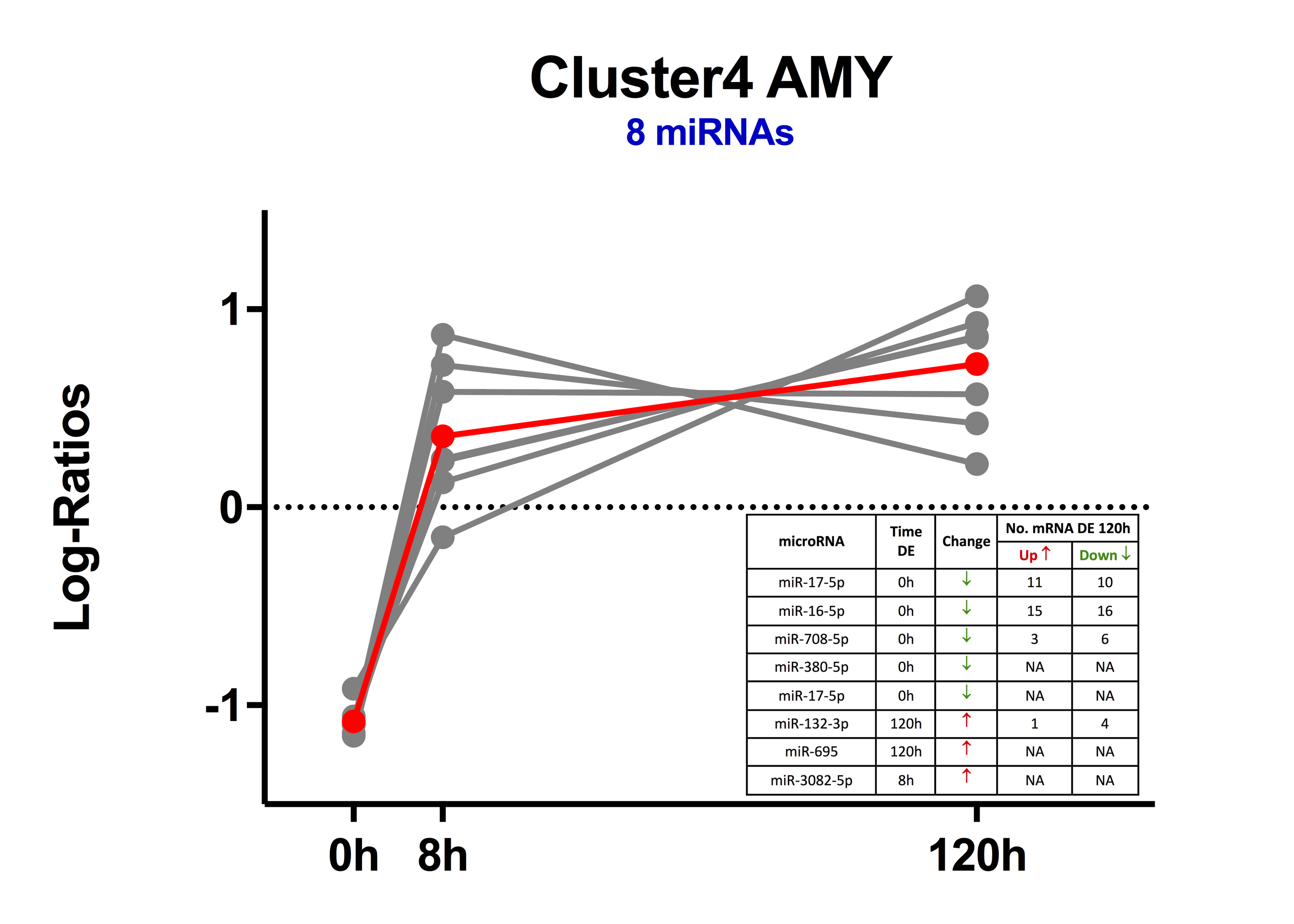

Supplement: S5 Fig — Average expression is plotted in red, and individual microRNAs are plotted in gray. Inset table provides expression data for microRNAs in the cluster. Time ("Time DE") and direction ("Change") of change are given for each microRNA (⬆ = up-regulation; ⬇ = down-regulation). The number and direction of 120h DE targets are given for each microRNA. (TIF) [file pone.0190841.s005.tif]

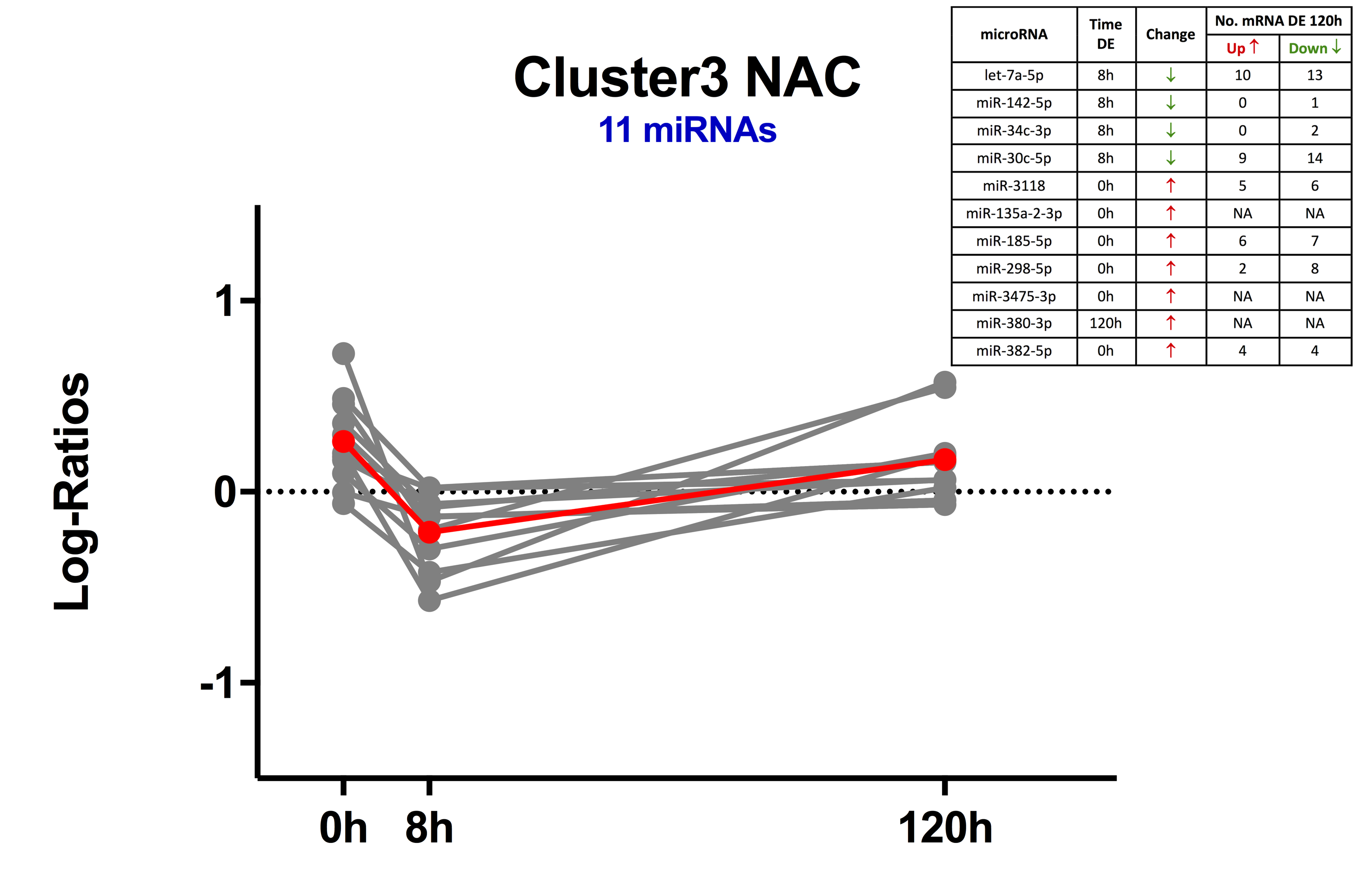

Supplement: S6 Fig — Average expression is plotted in red, and individual microRNAs are plotted in gray. Inset table provides expression data for microRNAs in the cluster. Time ("Time DE") and direction ("Change") of change are given for each microRNA (⬆ = up-regulation; ⬇ = down-regulation). The number and direction of 120h DE targets are given for each microRNA. (TIF) [file pone.0190841.s006.tif]

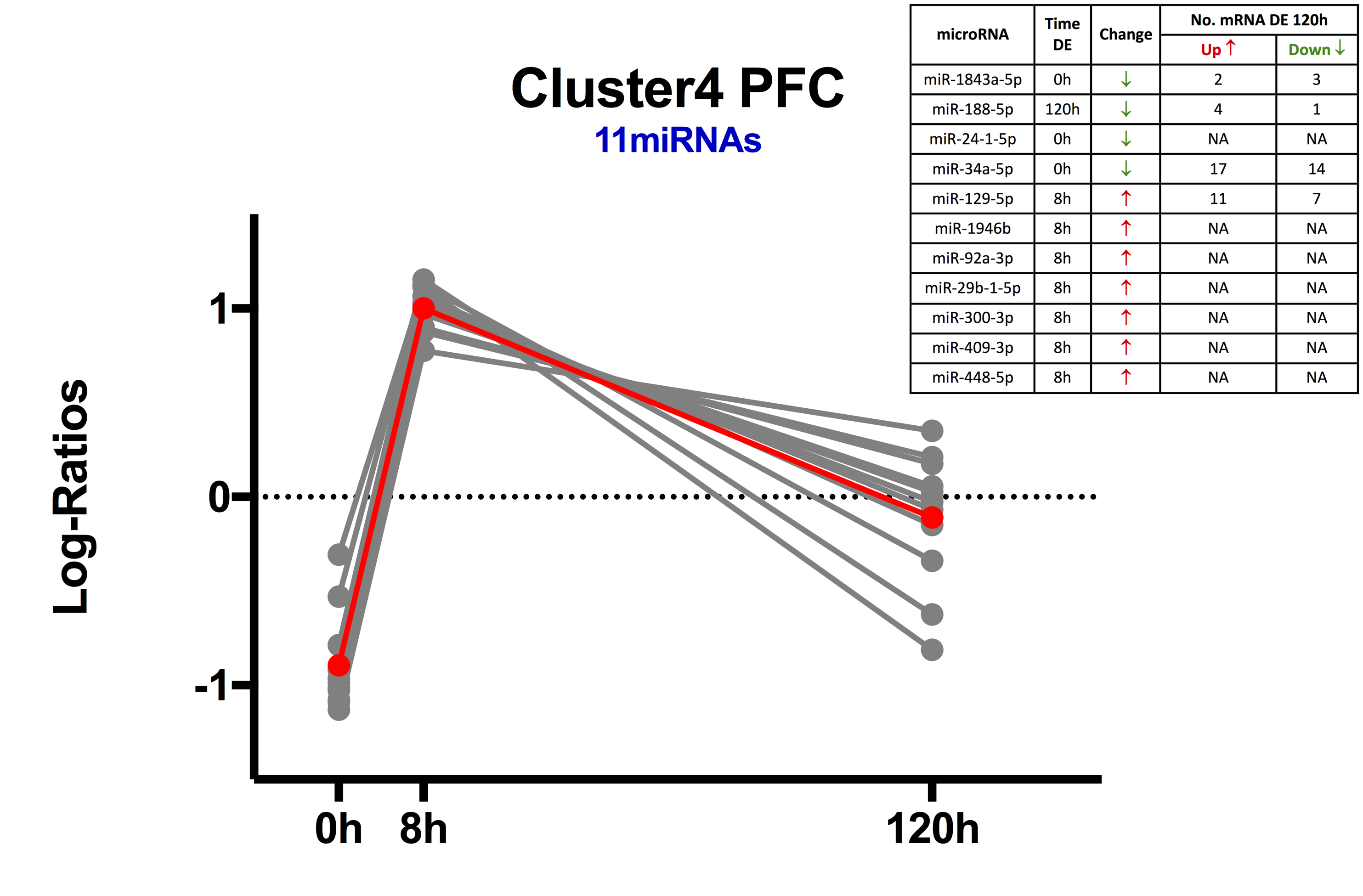

Supplement: S7 Fig — Average expression is plotted in red, and individual microRNAs are plotted in gray. Inset table provides expression data for microRNAs in the cluster. Time ("Time DE") and direction ("Change") of change are given for each microRNA (⬆ = up-regulation; ⬇ = down-regulation). The number and direction of 120h DE targets are given for each microRNA. (TIF) [file pone.0190841.s007.tif]
