# Supplementary material for: Long-term ethanol exposure: Temporal pattern of microRNA expression and associated mRNA gene networks in mouse brain
Source: PLoS One. 2018 Jan 9;13(1):e0190841. doi: 10.1371/journal.pone.0190841 (PMC5760035; doi:10.1371/journal.pone.0190841)
Supplement: S3 Fig — To investigate the potential time-dependent relationship of miRNA gene regulation, focused datasets were created from differentially expressed miRNAs (0 and 8 h only) and their differentially expressed target mRNAs (120h). For each of these miRNA-mRNA datasets, genes from the top three IPA-derived networks were merged and genes common to the merged networks were identified. This enabled us to focus on a key list of genes whose expression is modulated by miRNAs during withdrawal and remains dysregulated during protracted withdrawal, a period of time associated with drug relapse. (PPTX) [file pone.0190841.s003.pptx]

## Slide 1
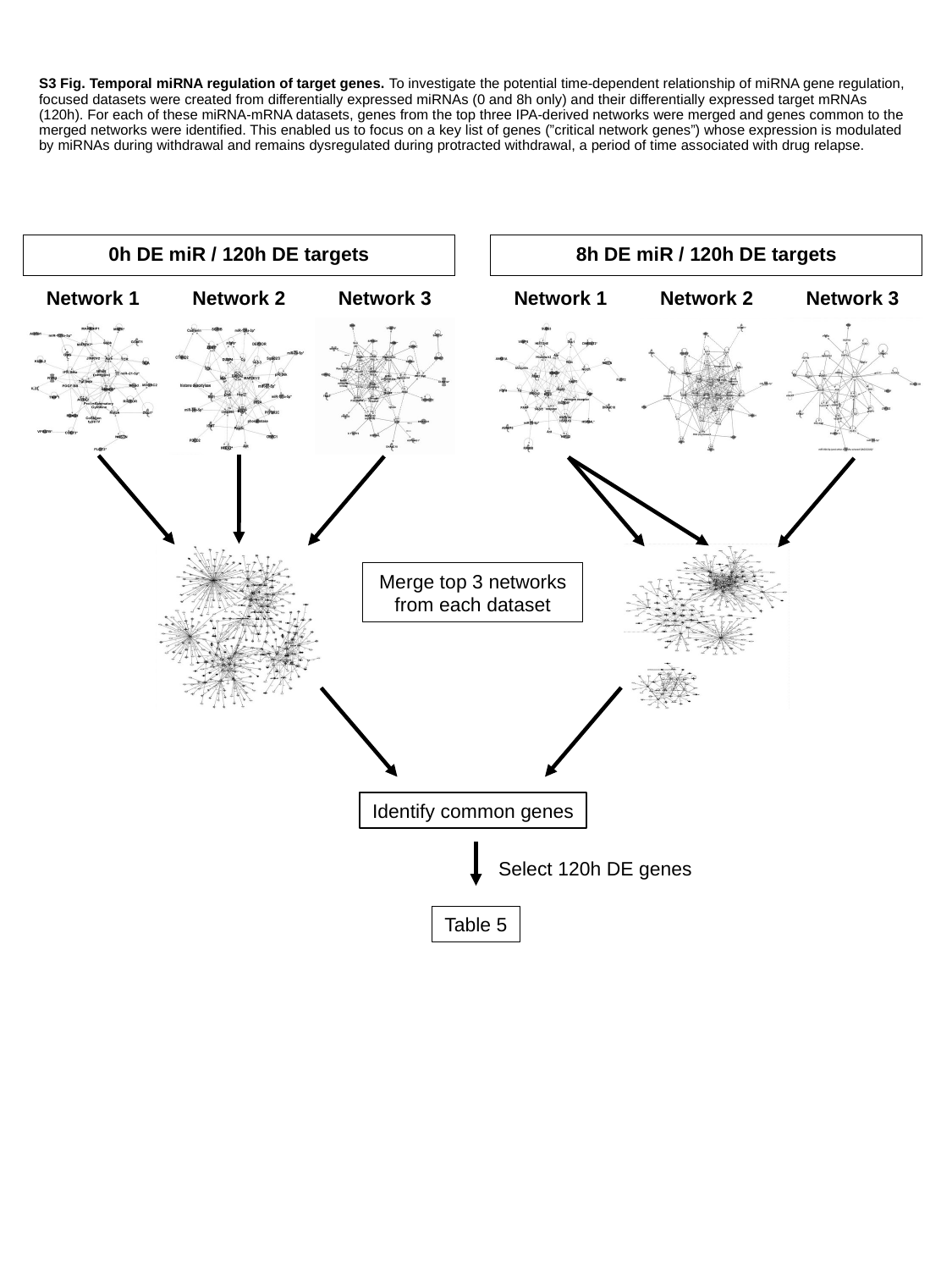

# S3 Fig. Temporal miRNA regulation of target genes. To investigate the potential time-dependent relationship of miRNA gene regulation, focused datasets were created from differentially expressed miRNAs (0 and 8h only) and their differentially expressed target mRNAs (120h). For each of these miRNA-mRNA datasets, genes from the top three IPA-derived networks were merged and genes common to the merged networks were identified. This enabled us to focus on a key list of genes (”critical network genes”) whose expression is modulated by miRNAs during withdrawal and remains dysregulated during protracted withdrawal, a period of time associated with drug relapse.
0h DE miR / 120h DE targets
8h DE miR / 120h DE targets
Network 2
Network 3
Network 2
Network 3
Network 1
Network 1
Merge top 3 networks from each dataset
Identify common genes
Select 120h DE genes
Table 5
