# Supplementary material for: Long-term ethanol exposure: Temporal pattern of microRNA expression and associated mRNA gene networks in mouse brain
Source: PLoS One. 2018 Jan 9;13(1):e0190841. doi: 10.1371/journal.pone.0190841 (PMC5760035; doi:10.1371/journal.pone.0190841)
Supplement: S1 Table — ND = not done. (DOCX) [file pone.0190841.s008.docx]

**S1 Table. Names of paired datasets used for discrete temporal analysis.** ND = not done.

|  | | **DE mRNA Targets** | | |
| --- | --- | --- | --- | --- |
|  |  | **0h** | **8h** | **120h** |
| **DE microRNAs** | **0h** | 0hDEmiR/0hDEtargets | 0hDEmiR/8hDEtargets | 0hDEmiR/120hDEtargets |
|  | **8h** | ND | 8hDEmiR/8hDEtargets | 8hDEmiR/120hDEtargets |
|  | **120h** | ND | ND | 120hDEmiR/120hDEtargets |
