# Supplementary material for: Long-term ethanol exposure: Temporal pattern of microRNA expression and associated mRNA gene networks in mouse brain
Source: PLoS One. 2018 Jan 9;13(1):e0190841. doi: 10.1371/journal.pone.0190841 (PMC5760035; doi:10.1371/journal.pone.0190841)
Supplement: S2 Table — MicroRNA families are derived from IPA and include all microRNAs with the same seed sequence. Colored cells identify microRNA families with multiple members dysregulated at 0h in PFC. (DOCX) [file pone.0190841.s009.docx]

**S2 Table. Multiple microRNAs differentially expressed at 0h in PFC are from the same family.** MicroRNA families are derived from IPA and include all microRNAs with the same seed sequence. Colored cells identify microRNA families with multiple members dysregulated at 0h in PFC.

| **Probe ID** | **miR Name** | **miR Family (IPA)** | **Fold Change (0h)** | **p Value (0h)** |
| --- | --- | --- | --- | --- |
| mmu-miR-101a_st | mmu-miR-101a-3p | miR-101-3p | **-1.49** | 1.81E-02 |
| mmu-miR-138-2-star_st | mmu-miR-138-2-3p | miR-138-2-3p | **-1.14** | 4.65E-02 |
| mmu-miR-200a_st | mmu-miR-200a-3p | miR-141-3p | **1.81** | 2.79E-02 |
| mmu-miR-141_st | mmu-miR-141-3p | miR-141-3p | **2.30** | 4.19E-02 |
| mmu-miR-155_st | mmu-miR-155-5p | miR-155-5p | **2.01** | 2.22E-03 |
| mmu-miR-182_st | mmu-miR-182-5p | miR-182-5p | **2.03** | 4.49E-03 |
| mmu-miR-183-star_st | mmu-miR-183-3p | miR-183-3p | **3.01** | 5.29E-03 |
| mmu-miR-183_st | mmu-miR-183-5p | miR-183-5p | **2.18** | 1.09E-02 |
| mmu-miR-1843-5p_st | mmu-miR-1843a-5p | miR-1843a-5p | **-1.33** | 1.31E-02 |
| mmu-miR-187_st | mmu-miR-187-3p | miR-187-3p | **1.61** | 1.65E-03 |
| mmu-miR-200b-star_st | mmu-miR-200b-5p | miR-200a-5p | **2.42** | 7.56E-03 |
| mmu-miR-200a-star_st | mmu-miR-200a-5p | miR-200a-5p | **1.72** | 4.63E-02 |
| mmu-miR-200c_st | mmu-miR-200c-3p | miR-200b-3p | **2.02** | 5.56E-03 |
| mmu-miR-200b_st | mmu-miR-200b-3p | miR-200b-3p | **2.08** | 1.07E-02 |
| mmu-miR-429_st | mmu-miR-429-3p | miR-200b-3p | **1.88** | 2.50E-02 |
| mmu-miR-211-star_st | mmu-miR-211-3p | miR-211-3p | **-1.95** | 3.06E-02 |
| mmu-miR-2137_st | mmu-miR-2137 | miR-2137 | **1.32** | 2.16E-02 |
| mmu-miR-24-2-star_st | mmu-miR-24-2-5p | miR-24-1-5p | **-1.22** | 2.60E-02 |
| mmu-miR-296-3p_st | mmu-miR-296-3p | miR-296-3p | **1.69** | 5.45E-03 |
| mmu-miR-30c-1-star_st | mmu-miR-30c-1-3p | miR-30c-1-3p | **1.51** | 3.11E-02 |
| mmu-miR-329_st | mmu-miR-329-3p | miR-329-3p | **-1.24** | 4.00E-02 |
| mmu-miR-344d_st | mmu-miR-344d-3p | miR-344d-3p | **-1.32** | 1.87E-02 |
| mmu-miR-34a_st | mmu-miR-34a-5p | miR-34a-5p | **-1.29** | 3.06E-02 |
| mmu-miR-421_st | mmu-miR-421-3p | miR-421-3p | **-1.38** | 1.69E-02 |
| mmu-miR-486-star_st | mmu-miR-486a-3p | miR-486-3p | **1.77** | 1.96E-02 |
| mmu-miR-3107-star_st | mmu-miR-486b-3p | miR-486-3p | **1.61** | 2.37E-02 |
| mmu-miR-149-star_st | mmu-miR-149-3p | miR-6967-5p | **-1.23** | 4.01E-02 |
| mmu-miR-7a-1-star_st | mmu-miR-7a-1-3p | miR-7-1-3p | **-2.09** | 7.77E-03 |
| mmu-miR-7a-2-star_st | mmu-miR-7a-2-3p | miR-7a-2-3p | **1.50** | 2.15E-03 |
| mmu-miR-7b-star_st | mmu-miR-7b-3p | miR-7a-2-3p | **1.32** | 4.56E-02 |
| mmu-miR-9-star_st | mmu-miR-9-3p | miR-9-3p | **-1.22** | 2.88E-02 |
